# Retraction: The C2238/αANP Variant Is a Negative Modulator of Both Viability and Function of Coronary Artery Smooth Muscle Cells

**DOI:** 10.1371/journal.pone.0324176

**Published:** 2025-05-09

**Authors:** The PLOS One Editors

After this article [1] was published, concerns were raised regarding results presented in Figs 2, 4-5, 7, S4-5, and S7-8. Specifically:

- There appear to be similarities between regions within the following panels:
  ◦ The Fig 2A CC2238/αANP panel.
  ◦ The Fig 5D β-Actin panel.
- The following panels or parts of panels appear similar:
  ◦ The Fig S7A CONTROL panel and the Fig 2A TT2238/αANP panel when flipped vertically.
  ◦ The Fig S7A CONTROL panel and the Fig 2A CONTROL panel when flipped vertically.
  ◦ The Fig S5A CC2238/αANP+siRNA1 panel, the Fig S7A mimic miR 21 panel, and the Fig S5A CC2238/αANP+siRNA2 panel when rotated 180°.
  ◦ The Fig S7A CC2238/αANP+mimic miR 21 panel and the Fig S8A CC2238/αANP+FSK panel.
  ◦ The Fig S4E β-actin panel and the Fig 4B β-actin panel.
- When levels are adjusted to visualize the background, there appear to be regions where the background around the bands does not appear to match the overall background of the panel in the p-eNOS panel of Fig 5C.

Regarding the cell migration image concerns in Figs 2A, S5A, S7A and S8A, the corresponding author stated that mistakes were made during data and figure preparation and that the original underlying images are no longer available. The corresponding author stated that the cell migration experiments were quantified under the microscope by counting the number of cells in different fields, but that the images in Figs 2A, S5A, S7A and S8A [1] are representative images not used for quantification. For the β-actin panels in Figs 4B and S4E, the corresponding author stated that the membrane used for Fig 4B was the same as that used for Fig S4E as the different molecular weights of the two proteins (Akt and eNOS) allowed the exposure to these antibodies on the same membrane containing the same experimental samples. The corresponding author stated that the underlying western blots for Figs 4B, 5C, 5D, 7D and S4E are no longer available.

In light of the above concerns that question the integrity and reliability of the published results, the *PLOS One* Editors retract this article.

SR agreed with the retraction. SM, FB, SDC, RS, MC, CB, SS, and MV either did not respond directly or could not be reached.

SR stands by the article’s findings.
